# NRP1 inhibition modulates radiosensitivity of medulloblastoma by targeting cancer stem cells

**DOI:** 10.1186/s12935-022-02796-4

**Published:** 2022-12-01

**Authors:** Manon Douyère, Caifeng Gong, Mylène Richard, Nadia Pellegrini-Moïse, Joël Daouk, Julien Pierson, Pascal Chastagner, Cédric Boura

**Affiliations:** 1 Université de Lorraine, CNRS, CRAN, UMR 7039, 54000 Nancy, France; 2 Department of Medical Oncology, National Cancer Center/National Clinical Research Center for Can-Cer/Cancer Hospital, Chinese Academy of Medical Sciences and Peking Union Medical College, Bei-Jing, 100021 China; 3 Université de Lorraine, CNRS, L2CM, UMR 7053, Campus Science, 54500 Vandœuvre-Lès-Nancy, France; 4 Service d’Onco-Hématologie Pédiatrique, CHRU-Nancy, 54000 Nancy, France

**Keywords:** Medulloblastoma, Neuropilin-1, Cancer stem cells, Radiotherapy

## Abstract

**Background:**

Medulloblastoma (MB) is the most common pediatric malignant brain tumor. Despite current therapies, the morbidity and recurrent risk remains significant. Neuropilin-1 receptor (NRP1) has been implicated in the tumor progression of MB. Our recent study showed that NRP1 inhibition stimulated MB stem cells differentiation. Consequently, we hypothesized that targeting NRP1 in medulloblastoma could improve current treatments.

**Methods:**

NRP1 inhibition with a novel peptidomimetic agent, MR438, was evaluated with radiotherapy (RT) in MB models (DAOY, D283-Med and D341-Med) in vitro on cancer stem-like cells as well as in vivo on heterotopic and orthotopic xenografts.

**Results:**

We show that NRP1 inhibition by MR438 radiosensitizes MB stem-like cells in vitro. In heterotopic DAOY models, MR438 improves RT efficacy as measured by tumor growth and mouse survival. In addition, clonogenic assays after tumor dissociation showed a significant reduction in cancer stem cells with the combination treatment. In the same way, a benefit of the combined therapy was observed in the orthotopic model only for a low cumulative irradiation dose of 10 Gy but not for 20 Gy.

**Conclusions:**

Finally, our results demonstrated that targeting NRP1 with MR438 could be a potential new strategy and could limit MB progression by decreasing the stem cell number while reducing the radiation dose.

**Supplementary Information:**

The online version contains supplementary material available at 10.1186/s12935-022-02796-4.

## Background

Medulloblastoma is (MB) is a serious childhood malignant brain tumor, accounting for approximately 20% of all pediatric central nervous system tumors. Craniospinal irradiation is usually one of the most important effective therapies combined with surgical excision and chemotherapy [1]. However, these combined therapeutic approaches are mainly non-specific and aggressive, inducing severe damage to the developing brain, especially for young patients [2]. Moreover, approximately one-third of the patients still die due to tumor recurrence [3]. It is urgent to find new therapies, especially for patients with high risk of recurrence.

The identification of four MB subgroups (WNT, SHH, Group 3, and Group 4) based on their respective molecular characteristics has provided a better understanding of tumor development and progression [4–6]. Recently, features of the subgroups revealed that each contains variant subtypes that helped risk stratification of MB patients [7, 8]. TP53 mutated SHH subgroup as well as subgroup 3 with MYC amplification have the worst prognosis with a 5-year survival rate  < 50% [9]. Tumor heterogeneity is also considered an important prognostic factor, which indicates that a small special cell population could survive after chemoradiotherapy and induce recurrences. It has been suggested that cancer stem cells (CSCs) are probably at the origin of the tumorigenesis as previously shown in medulloblastoma patients [10–12], and may be involved in resistance to therapy and recurrence [13–16]. Indeed, Blazek et al*.* found that CD133 + MB cells were more radioresistant than CD133- MB cells [17]. The identification of new molecular targets specific for MB stem cells is a major challenge to improve the management of this pathology. Human MBs are propagated by cells expressing the progenitor marker CD15/SSEA [18]. CD15 is a carbohydrate antigen that is expressed on both progenitors and stem cells in the embryonic and adult central nervous systems [19, 20]. CD15 is considered an important marker for medulloblastoma propagating cells, also named medulloblastoma stem-like cells [18, 21].

Neuropilin-1 (NRP1) has the functions of development of neuronal and vascular systems [22, 23], which is known as a co-receptor by complexation with other transmembrane receptors such as VEGFR [24, 25]. The overexpressed of NRP1 in various cancers, including MB, has been reported to has been correlated with poor prognosis with upregulation of cell proliferation or migration [26–28]. Indeed, our previous work showed that NRP1 was overexpressed in MB and related to the undifferentiated status of MB and that a novel NRP1 inhibitor (MR438) can stimulate the differentiation of MB stem-like cells [29]. As CSCs are related to radioresistance, the differentiation induced effect might enhance the efficiency of RT with MR438 as a radiosensitizer, to decrease tumor growth, control recurrence, and limit brain damage due to a lower dose of RT. Tuftsin (TKPR: Thr-Lys-Pro-Arg) is a natural peptide antagonist of NRP-1 was used in our work to compare the effect of MR438 [29, 30]. In our work, we hypothesize that NRP1 inhibition with this new peptidomimetic compound could improve radiotherapy treatment by targeting medulloblastoma stem-like cells, and we evaluated the effect of NRP1 inhibition with MR438 concomitant with RT in 3 subgroup MB models (DAOY, D283-Med and D341-Med) by in vitro experiments on stem-like cells, as well as on in vivo heterotopic and orthotopic xenografts.

## Materials and methods

### Cell culture and drug solutions

The MB cell lines of DAOY, D283-Med and D341-Med were purchased from ATCC cell biology collection (Manassas VA, USA). MB stem-like cells cultures were grown in DMEM/F12 medium (Gibco, Life Technologies Corporation, UK) containing B27 and N2 supplement (Gibco, LifeTechnologies Corporation, USA), 40 µg/ml heparin, 1% insulin, 20 ng/mL human recombinant epidermal growth factor (EGF) and basic fibroblast growth factor (bFGF) (EGF and FGF from Miltenyi Biotec, Germany). After a 3-day culture in hydrophobic flasks at 37 °C with 5% CO_2_ in a humidifier atmosphere, spheres were obtained. MB stem-like cells were dissociated from spheres using Accumax (Gibco, Life Technologies Corporation, UK) and seeded in 25 cm^2^ flasks depending on the experiment.

For in vitro experiments, the stem like cells were exposed to MR438 [31] (molecular weight: 527.20 g/mol, supplied by L2CM-UMR 7053 in powder form) and the natural ligand (tetrapeptide: TKPR) known to target NRP1 named tuftsin [30, 32] (molecular weight: 500.60 g/mol, BACHEM, Switzerland) at 25 µmol/L for 72 h. MR438 and tuftsin were each dissolved in PBS and stored in aliquots at −20 °C.

### Radiosensitivity of medulloblastoma stem like cells exposed to MR438

RT was performed using a X-RAD 320 Irradiator (Precision X Ray, USA). For in vitro radiosensitivity, MB stem cells were obtained as previously described. MB cells were seeded onto 6 well plates (20 000 cells/well for DAOY-MS and 50 000 cells/well for D283-MS and D341-MS) were suspended in 2 ml of DMEM/F12 containing 1% methylcellulose (SIGMA, USA). Cells were pretreated with MR438 and tuftsin (25 μM) for 72 h before RT. Cells were then exposed to a 15 × 15 cm^2^ radiation field with doses of 0, 2, 4, 6, and 10 Gy at room temperature. Following irradiation, all cell samples were returned to a 5% CO_2_ incubator for 2 weeks. Then, cell colonies were incubated with 0.5% MTT solution (thiazolblue tetrazolium bromide, 98%, Acros Organics^™^), and colonies larger than 30 μm in diameter were quantified using GelCount^™^ (Oxford Optronix, UK). Each experiment was repeated 6 times with 3 independent wells. From data, different radiosensitivity parameters were calculated such as SF2 (survival fraction at 2 Gy) and DMF2 (dose modified factor at 2 Gy) and alpha value. SF2 is calculated by using 0 Gy as reference, DMF2 is the ratio of the dose required to obtain SF2 in the presence of MR438 at 2 Gy. The alpha values were obtained by fitting a linear quadratic model to the clonogenic assay curves with the different irradiation doses. Alpha value represents the intrinsic radiosensitivity of the irradiated cells: cells with a higher alpha are more sensitive to radiation.

### Animal models

Immunodeficient mice (NMRI-nu, Janvier Labs, France) xenografted heterotopically with MB cells were used within the agreement of the French Minister of Research (agreement n°APAFiS #8731). DAOY, D283-Med and D341-Med cells were re-suspended in PBS, and mixed 1:1 with Matrigel (BD Biosciences). The cells (2 × 10^6^ cells/200 μL) were injected subcutaneously into the right and left flanks of 6-week-old nude mice. When tumors reached a volume of 1000 mm^3^, the tumors were divided into small pieces of 2–3 mm^3^ and re-planted subcutaneously in the inguinal region near the femoral vessel of mice. When palpable tumors reached a volume of 250 mm^3^, the mice were subjected to radiation as described below. Tumor size was monitored every 2 days by measuring two dimensions and the volume was calculated by calculating (length × width^2^)/2. Tumor growth was normalized by taking tumor volume at the start of treatment as a reference. The mice were followed for at least 60 days or until the tumor size reached approximately 1000 mm^3^ (end point). Mice were then euthanized, and the tumors were harvested for the other experiments.

Orthotopic xenograft models in nude mice (NMRI-nu, Janvier Labs, France) were used within the agreement of the French Minister of Research (agreement nAPAFiS #20085). Mice were anesthetized by intraperitoneal injection (4 µL/g of weight) of solution mixture of xylazine/ketamine (90 and 8 mg/kg, respectively). The heads of the mice were fixed in a stereotaxic instrument (KOPF^®^). DAOY-luc cells (Lentivirus-LV-CMV-Firefly luciferase (CMV, puromycin)) were prepared from fresh culture to ensure optimal viability. MB cells (cell suspension: 1.10^5^ cells/µL) were stereotaxically implanted into the cerebellum by using the following coordinates: 2 mm right and bottom to the lambda suture and 2 mm ventral from the surface of the skull. Cell suspensions were injected by HAMILTON syringe with an infusion rate of 0.5 µl/min for 5 µL total. Twenty-one days following tumor transplantation, the animals were randomly divided into four groups: control (CTL), MR438, radiotherapy (RT) and RT + MR438.

Tumor growth was followed by bioluminescence imaging using OptiMAX (Precision X-Ray Inc, North Brandford, CT) once a week. An intraperitoneal injection was carried out to administer a solution of D-Luciferin (D-Luciferin Firefly, PerkinElmer^®^ #122799) diluted in a sterile PBS solution (at 15 mg/ml and 10 µL/g body weight). Then, the animals were anesthetized with an intraperitoneal injection a xylazine/ketamine mixture (90 and 8 mg/kg, respectively). Mice are imaged, positioned on their backs, 15–20 min after the luciferin injection. The intensity of bioluminescence was determined with Image J^®^ software by drawing a constant region of interest over the tumor regions and measuring the intensity of the signal in number of total photons/areas.

### In vivo combined therapy using radiotherapy and NRP1 inhibition

For tumor RT in vivo, animals were anesthetized with a mix of xylazine/ketamine (90 and 8 mg/kg, respectively) and positioned such that the apex of each flank tumor was at the center of a 3 × 3 cm^2^ or 1 × 1 cm^2^ radiation field for heterotopic or orthotopic xenograft treatment, respectively, with the rest of the mice shielded from radiation by using the protective baffle. Tumors were exposed to RT at a dose of 2 Gy per fraction for 5 days (D1–D5) with/without MR438 or tuftsin administration. Molecular treatments were carried out 2 h before RT with 2 I.V. administrations at D1 and D3 at a dose of 10 mg/kg (4 µL/g body weight) for the heterotopic model, and with 3 I.V. administrations at D1, D3 and D5 at a dose of 10 mg/kg (4 µL/g body weight) for the orthotopic model, for 1 or 2 weeks.

### Medulloblastoma stem like cells harvested from tumors

When the heterotopic tumor volume reached 1000 mm^3^, the mice were sacrificed, and the tumors were harvested for dissociation. A part of the tumor was separated into 1–2 mm^3^ small pieces, and digested for 75 min in 7.65 ml dissociation buffer including HBSS (6 mL, Hank’s Balanced Salt Solution, Sigma, USA), collagenase type IV (0.6 WU/mL, Gibco, USA), Dispase II (1 mg/mL, Sigma, USA), DNAse I (200 U/mL, Roche, Germany), CaCl2 (75 μM) and MgCl2 (125 μM). Buffer DNAse I (200 U/mL) was then used to remove DNA interference with other living cells so that the cells do not aggregate easily. Cells were re-suspended in HBSS after eliminating the red blood cells by lysis buffer of NHCl4 (0.15 M), KHCO3 (10 mM), and EDTA (100 μM). Cell suspensions were then rapidly stained with the same volume of 0.4% trypan-blue solution and deposited in counting chamber slides (TC20, Biorad). The number of surviving cells was counted twice before the cells were seeded for clonogenic assays. Cells were seeded into 6 well plates at a density of 100 000 cells/well for DAOY tumors and 200 000 cells/well for D283-Med tumors and D341-Med tumors and cultivated for 2 weeks in serum free condition. Viable medulloblastoma stem cells were evaluated by metabolic activity (MTT assay). For each tumor, the experiments were performed in 3 independent wells considered as a single statistical value.

### Histological analysis

All tumors were collected and kept within a 4% formaldehyde solution for 48 h (1 week for brain tumors). Then, the samples were transferred into an inclusion cassette within a 70% alcohol solution. The cassettes were transferred to an automated dehydration machine (HISTO-PRO 300) overnight for paraffin inclusion. A microtome (LEICA RM 2135) was used to cut the paraffin blocks into 5 µm thick slices. Then, the sections were fixed over the slides for staining. The staining step was achieved either by a manual procedure or an automated machine with a fixed protocol. H&E staining was used for microscopic observations.

Samples were incubated with primary antibodies against Ki67 (Abcam, ab16667, dilution of 1/1500), NRP1 (Abcam, ab81321, dilution of 1/1500), CD15 (Sigma-Aldrish, SAB5500041, dilution of 1/500) and ColIV (Novotec, dilution 1/3200). A Nikon Eclipse E600 equipped with a camera microscope (Nikon–DS Fi1) was used to take pictures. The histological sample slides were analyzed with the free QuPath software by using the vector staining tool to count the total number of cells by staining cell nuclei with hematoxylin and the positive cells expressing the interest proteins by staining with the brown chromogen DAB (3,3ʹ-Diaminobenzidine).

### Gene expression of stem cell markers by qRT-PCR

The gene expression of stem cell markers (Table 1) was analyzed by quantitative reverse transcription PCR (qRT-PCR). After extraction with an All Prep-DNA RNA-Mini Kit (Omega), Total RNA was reverse-transcribed with the iScript^™^ cDNA synthesis Kit (BioRad) following the manufacturer’s protocol. Real-time PCR was performed with SyberGreen PCR Supermix (BioRad) using the CFX96 Real-Time System (BioRad). The qPCR conditions were 95 °C for 2 min and 39 cycles of 95 °C for 5 s and 63 ~ 68 °C for 30 s, and the hybridization temperature was dependent on the primers (Table 1). All values were normalized to RPL13A and TBP, and the ΔΔCt method was used to estimate the fold change expression over control samples.

**Table 1:** Sequences and annealing temperatures of primers used in qRT-PCR

Gene	Primer sequence (5′–3′)	Tm (°C)
CD133	Fwd: TCCGGGTTTTGGATACACCCTA	68 °C
	Rev: CTGCAGGTGAAGAGTGCCGTAA	
Sox 2	Fwd: TTTCACGTTTGCAACTGTCC	63 °C
	Rev: AGTCTCCAAGCGACGAAAAA	
RPL13A	Fwd: GTTCCTGCTGCCCTCAAG	60 °C
	Rev: GTCACTGCCTGGTACTTCC	
TBP	Fwd: GAGCTGTGATGTGAAGTTTCC	60 °C
	Rev: TCTGGGTTTGATCATTCTGTAG	

### Statistical analysis

All results are given as mean ± standard error of the mean (SEM). Nonparametric Mann–Whitney test and Student’s T-test for two-by-two comparisons were employed to determine the statistical significance using GraphPad Prism (GraphPad Prism 8.0, USA) with a minimum of 6 repetitions for in vitro and in vivo experiments. For all figures, p < 0.05 was considered significant. For in vivo experiments, the time growth of tumors to reach 1000 mm^3^ (set end point) served to build the Kaplan–Meier curves and log-rank test was used to compare survival curves.

## Results

### Radiosensitivity of medulloblastoma stem like cells to MR438

**Fig. 1:**
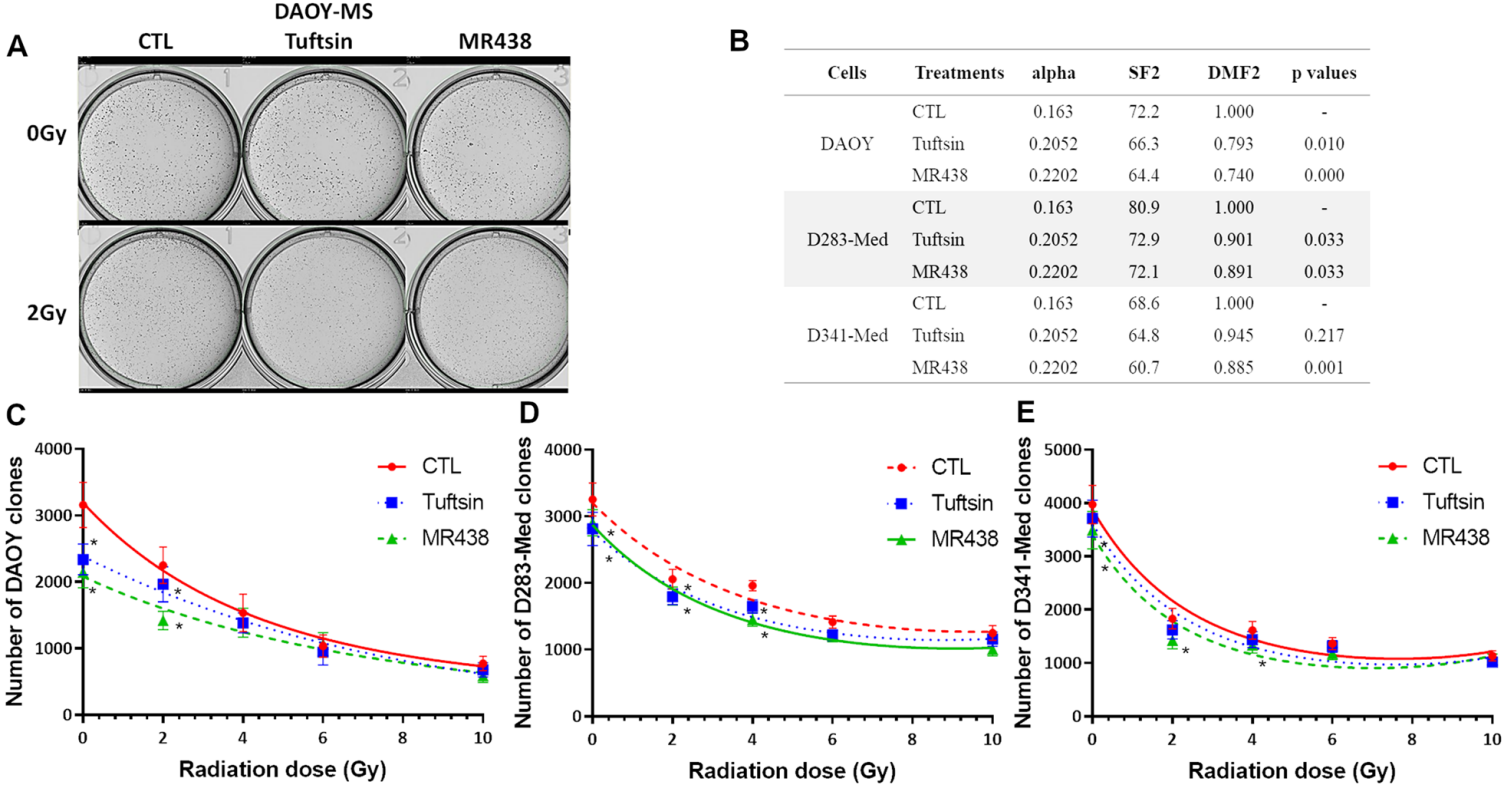
Effect of MR438 on the in vitro radiosensitivity of medulloblastoma stem-like cells. **A** Representative images of the colony formation assay using methylcellulose for DAOY-MS treated with tuftsin and MR438 at 0 and 2 Gy. **B** Tumor radiosensitivity factors: alpha, SF2 and DMF2 were calculated after exposition to tuftsin and MR438 from fitting curves **C**, **D**, **E**; the significance for DMF2 and each point of fitting curves were given for each condition in comparison to CTL. **C**, **D**, **E**. Representation of clone numbers after irradiation at 2, 4, 6 and 10 Gy (1*10^5^ cells/well for DAOY-MS and 2*10^5^ cells /well for D283-MS and D341-MS). *MS* Medullosphere, *SF2* Surviving fraction at 2 Gy, *DMF2* Dose modified factor at 2 Gy. *p < 0.05 versus 0 Gy, n = 6

The effect of MR438 on radiosensitivity of medulloblastoma stem-like cells was assessed by using the colony formation assay (Fig. 1). Cells were pre-treated with 25 µM tuftsin and MR438 for 72 h before RT exposure. After irradiation, the number of clones was evaluated. For DAOY-MS cells, the number of clones was significantly decreased after exposure to NRP1 targeting compounds at 2 Gy, especially after exposure to MR438 (Fig. 1A, B and C). D341-Med cells were the most sensitive to irradiation, but MR438 still had a radiosensitive effect at 2 Gy (Fig. 1E). It is interesting to observe a reduction of approximately 25% of DMF2 (dose modified factor at 2 Gy corresponding to the relative dose of irradiation required for a given effect in the drug-treated group as compared with a radiation-only group) for DAOY and approximately of 10% for D283-Med and D341-Med stem cells (Fig. 1B).

### Radiosensitivity of heterotopic medulloblastoma models with MR438

**Fig. 2:**
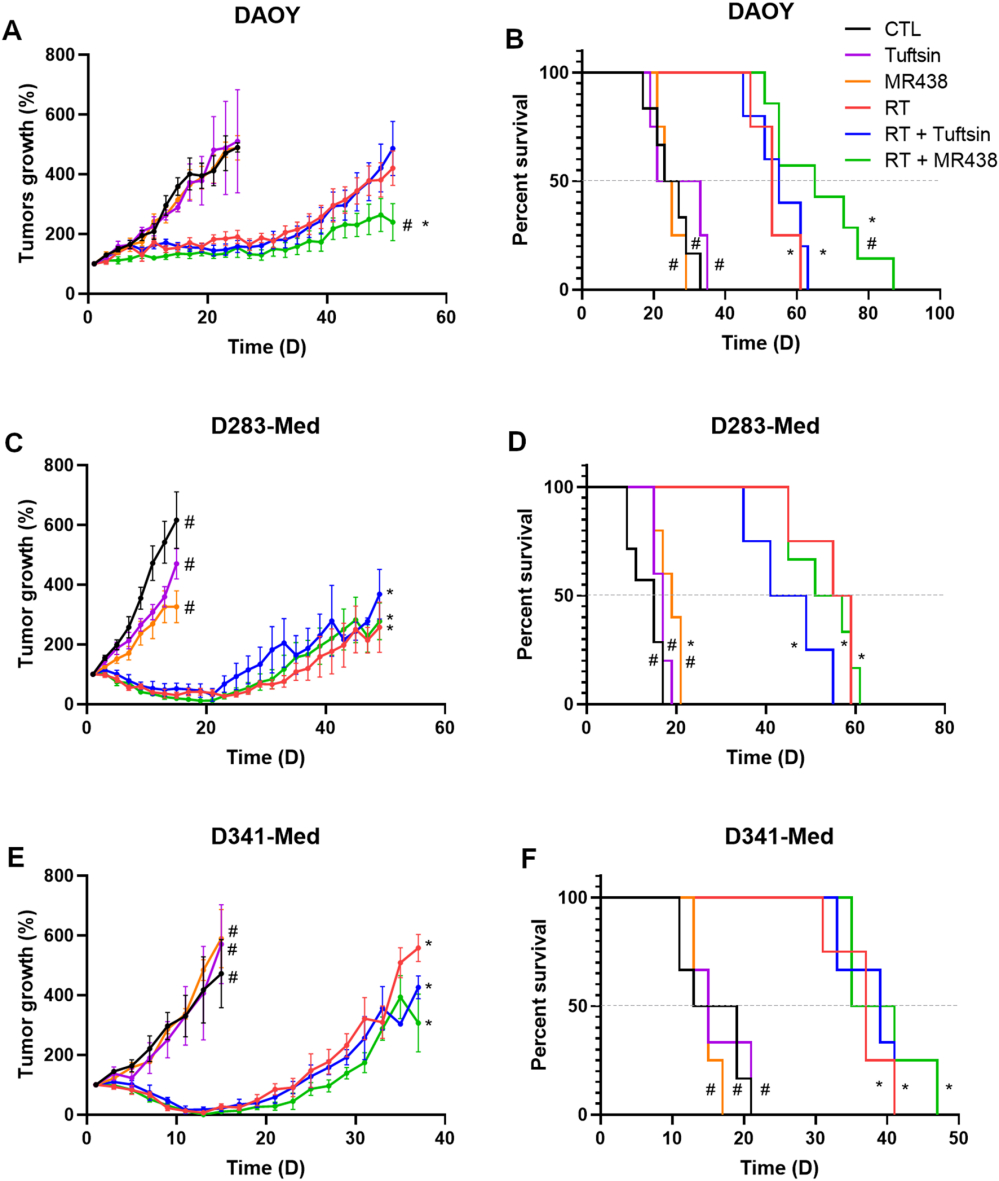
Effect of MR438 on tumor growth and mouse survival after irradiation with heterotopic xenografts. The percentage of tumor growth of DAOY **A**, D283-Med **C** and D341-Med **E** after radiation (5 × 2 Gy) and/or administration of MR438 or tuftsin (10 mg/kg) was calculated from tumor size before treatment as a reference. Survival fractions were represented for DAOY **B**, D283-Med **D** and D341-Med **F** using the maximal tumor size of 1000 mm^3^ as endpoint. *p < 0.05 versus CTL and #p < 0.05 versus RT, n = 6

Based on the results of our previous work [29] and of in vitro radiation assays, heterotopic xenograft models were used to confirm whether MR438 could also improve the radiosensitivity of MB in vivo. MR438 alone (Fig. 2) had no significant effect on tumor growth and mouse survival with DAOY and D341-Med tumors, but it seemed to have a significant effect on the survival of mice bearing D283-Med tumors. When combined with RT, MR438 decreased the tumor growth of DAOY compared to RT alone or compared to RT + Tuftsin (Fig. 2A). Similarly, the survival of mice bearing DAOY tumors treated with RT + MR438 was significantly improved, with a median survival of 65 days compared to that of mice treated with RT alone (53 days). D283-Med and D341-Med tumors are more sensitive to fractionated irradiation at the beginning of the treatment according to our in vitro results. Despite a median survival improvement of 3 and 1 days respectively, for D283-Med and D341-Med tumors, the combination of RT with MR438 did not significantly improve the radio-sensitivity of these tumors.

### Effect of combined RT and MR438 on NRP1 expression in heterotopic medulloblastoma models

**Fig. 3:**
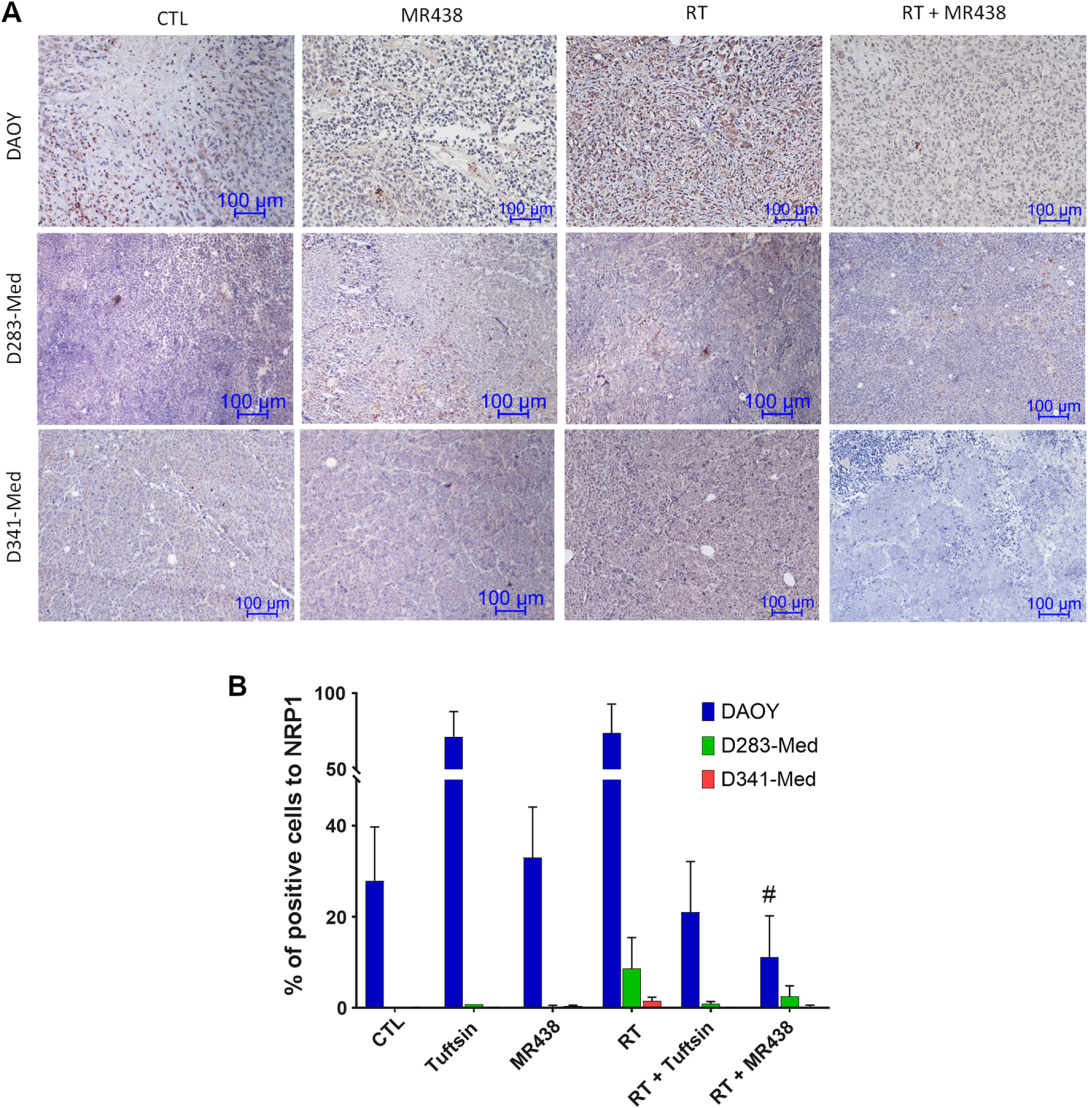
Effect of MR438 on NRP1 expression in heterotopic xenografts in nude mice. Representative images of NRP1 expression in tumors after irradiation and/or MR438 treatments **A**; scale bars represent 100 µm. The percentage of cells positive for NRP1 **B** for each condition was calculated with QuPath open software from approximately 5000 cells for 3 different images with wide field of observation from 3 different tumors. The analysis were performed at the experimental endpoint (corresponding to an average of 19 ± 3 days for mice of non-irradiated groups and 51 ± 8 days for mice of irradiated groups). *p < 0.05 versus CTL and #p < 0.05 versus RT; n = 6

To evaluate the effect of MR438 on the expression of NRP1 immunohistochemistry analysis of tumors after treatments was performed. DAOY tumors more strongly expressed NRP1 protein than D283-Med or D341-Med (Fig. 3A and B). For DAOY tumors treated with MR438 combined with RT, NRP1 protein expression decreased significantly compared to RT alone. Strangely, tuftsin or RT alone increased NRP1 protein expression in DAOY tumors. For D283-Med or D341-Med tumors, no effect was observed on NRP1 protein expression. Moreover, the mRNA expression of NRP1 was also evaluated, and no change was noted regardless of the treatment conditions or the tumor model (data not shown). We studied the expression of Ki67, a proliferation marker, by immunohistochemical analysis of tumors to assess the effect of our treatment on cell proliferation within the tumors (Additional file 1: Figure S1). No effect was observed on Ki67 protein expression for D283-Med and D341-Med in comparison to the CTL group or the RT group (Additional file 1: Figure S1B). Interestingly, the Ki67 protein expression was significantly decreased in the RT + MR438 group compared to the RT group for DAOY tumors (Additional file 1: Figure S1B).

### Effects of combined RT and MR438 on medullosphere formation and the expression of stem cell markers from heterotopic medulloblastoma models

**Fig. 4:**
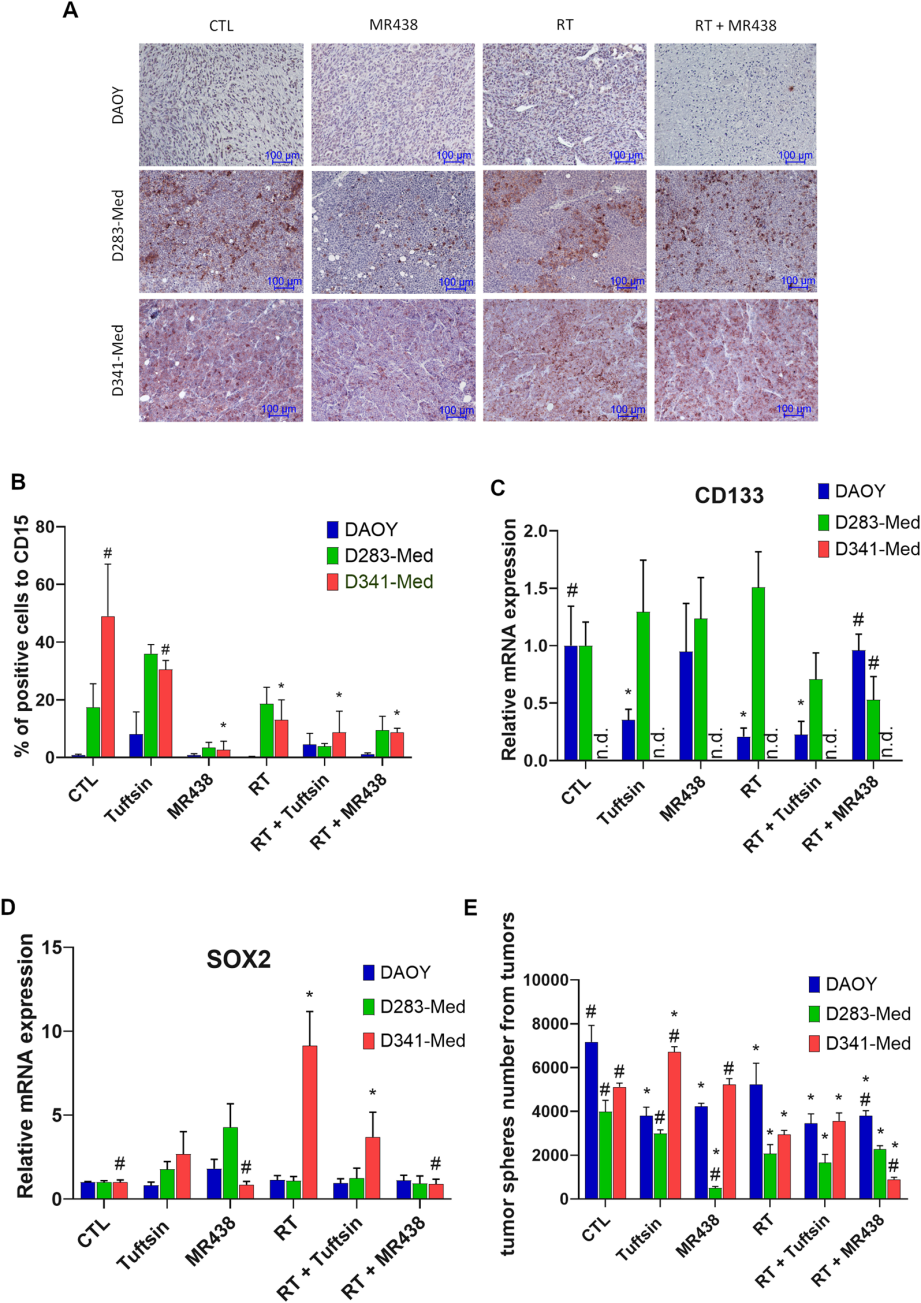
Effect of MR438 on the expression of stem cell markers and tumors spheres number of medulloblastoma stem-like cells after tumors dissociation. Representative images of CD15 expression in tumors after irradiation and/or MR438 treatments **A**; scale bars represent 100 µm. The percentage of cells positive for CD15 **B** was calculated with QuPath open software from approximately 5000 cells for 3 different images with wide field of observation from 3 different tumors. The gene ex-pression of CD133 **C** and Sox2 **D** was analyzed by quantitative reverse transcription PCR (qRT-PCR). Tumor spheres number of medulloblastoma stem cells cultivated in serum free condition after tumors dissociation of DAOY, D283-Med and D341-Med tumors **E**. The analysis were performed at the experimental endpoint (corresponding to an average of 19 ± 3 days for mice of non-irradiated groups and 51 ± 8 days for mice of irradiated groups). *p < 0.05 versus CTL and #p < 0.05 versus RT, n = 6

To confirm the effects of NRP1 inhibition with MR438 on medulloblastoma stem cells, we evaluated the expression of stem cell markers (CD15, CD133 and Sox2) as well as the self-renewal ability of medulloblastoma cells to form medullospheres after tumor dissociation (Fig. 4). CD15, as a CSCs marker for MB, was observed in less than 1% of DAOY cells while it was found in 17 and 48% of D283-Med and D341-Med cells, respectively. MR438 significantly decreased the expression of CD15 protein in D341-Med tumors; nevertheless, mRNA expression was not modified by the different treatment conditions (data not shown). mRNA expression of other markers of stem cells, such as CD133 and Sox2, has been studied. MR438 alone and RT + MR438 significantly decreased Sox2 mRNA expression for D341-Med but not for the other cell lines (Fig. 4D). A decrease in CD133 was shown with treatment association only for D283-Med (Fig. 4C). Interestingly, RT + MR438 strongly and significantly decreased the clone number of medulloblastoma stem cells from DAOY, and D341-Med tumors (Fig. 4E).

### MR438 is effective at a low cumulative RT dose in orthotopic medulloblastoma models

**Fig. 5:**
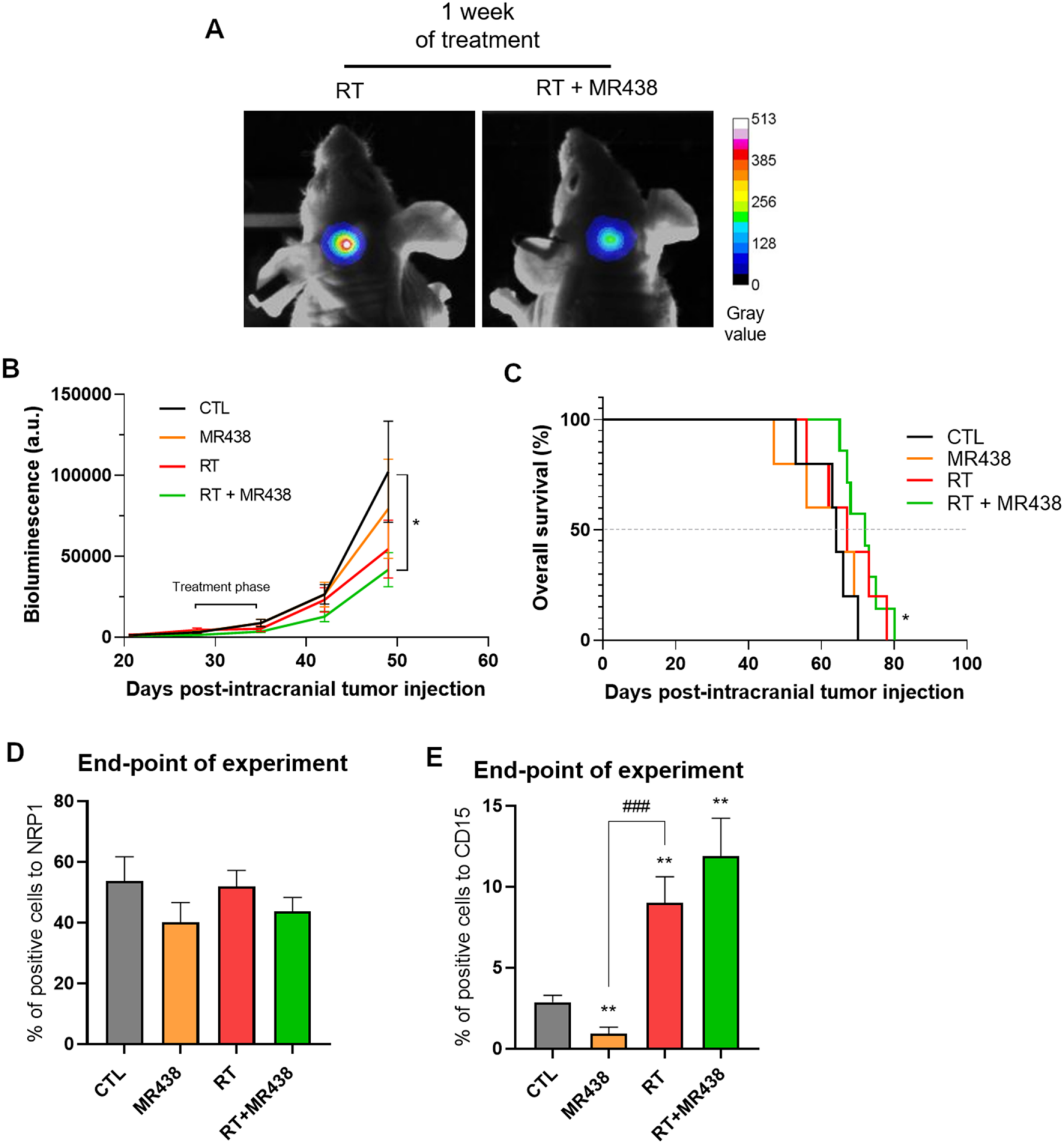
Effect of MR438 in combination with RT on intracerebellum DAOY growth and survival fraction after one week of treatment. Intracerebellum tumor visualized by in vivo bioluminescence imaging on day 50 post-tumor cell implantation for the RT group vs the RT + MR438 group after one week of treatment (**A**). Tumor growth after one week of treatment (radiations: 5 × 2 Gy and/or administra-tion of MR438: 10 mg/kg) **B** and survival fraction **C** were evaluated by bioluminescence. The percentage of cells positive for NRP1 **D** or CD15 **E** at the end point of the experiment was quantified with QuPath open software from approximately 5000 cells for 3 different images with wide field of observation from 3 different tumors. *p < 0.05 versus CTL, **p < 0.01 versus CTL, ###p < 0.001 versus RT; n = 7 per group for tumor growth and n = 4 per group for histological analysis

**Fig. 6:**
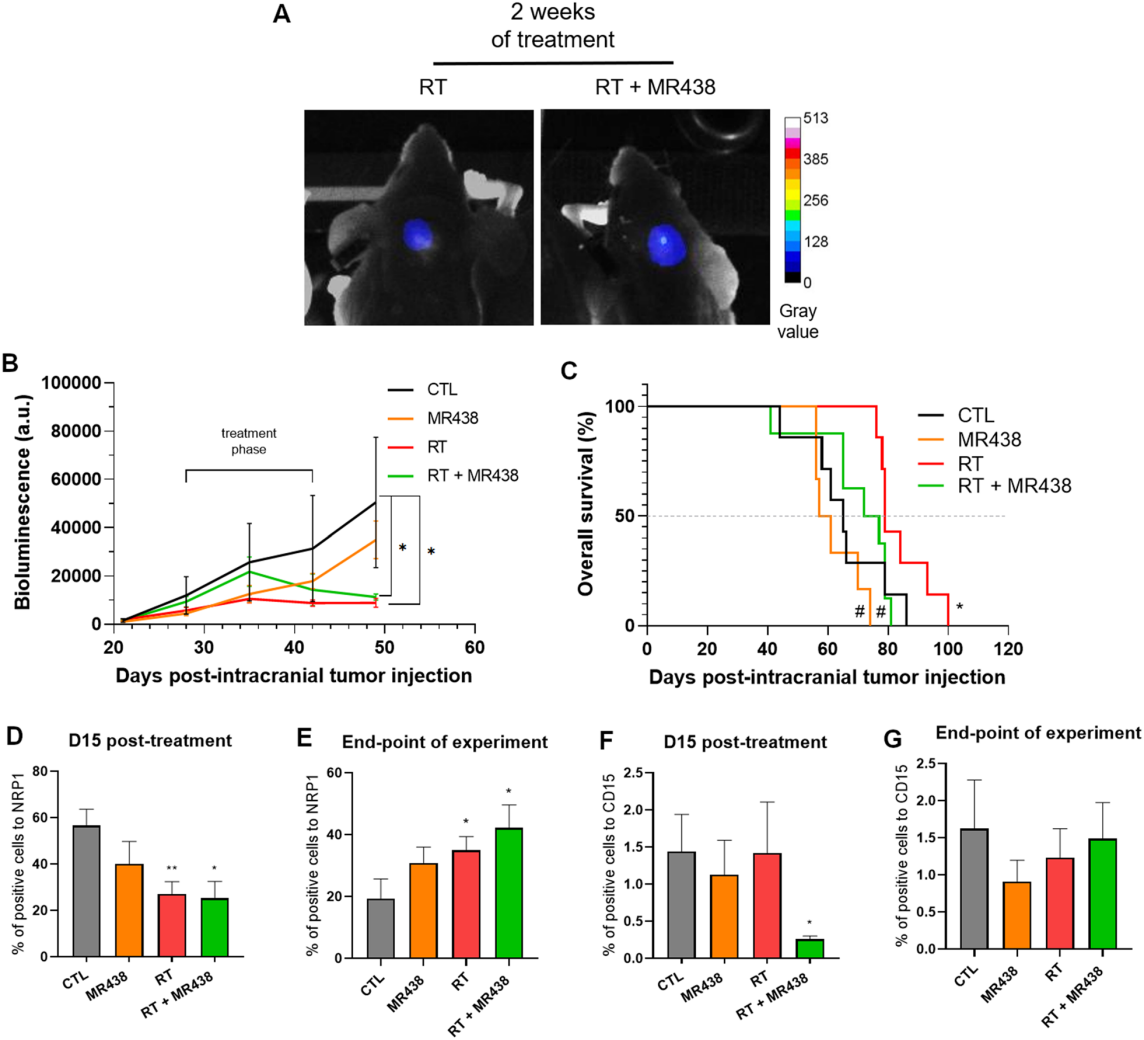
Effect of MR438 in combination with RT on intracerebellum DAOY growth and survival fraction after two weeks of treatment. Intracerebellum tumor visualized by in vivo bioluminescence imaging on day 50 post-tumor cell implantation for the RT group vs the RT + MR438 group (**A**). Tumor growth after two weeks of treatment (radiations: 10 × 2 Gy and/or administration of MR438: 10 mg/kg) **B** and survival fraction **C** were evaluated by bioluminescence. The percentage of cells positive for NRP1 **D**, **E** or CD15 **F**, **G** at 15 days post-treatment **D**, **F** or at the end point of the experiment **E**, **G** was quantified with QuPath open software from approximately 5000 cells for 3 different images with wide field of observation from 3 different tumors. *p < 0.05 versus CTL, **p < 0.01 versus CTL, #p < 0.05 versus RT; n = 7 per group for tumor growth and n = 3 per group for histological analysis

The effects of NRP1 inhibition in combination with radiotherapy were studied in an orthotopic medulloblastoma model by using medulloblastoma cells expressing luciferase transplanted into the right cerebellum. Mice were treated for 1 or 2 weeks (5 × 2 Gy per week), observed daily, and sacrificed at the first clinical symptom. In the case of 1 week of treatment (Fig. 5), a significant slowdown in tumor growth was observed for the RT + MR438 group compared to the nontreated group. The median survival (Fig. 5B) was significantly improved for the RT + MR438 group as compared to the control group, contrary to the RT group (72 days for RT + MR438, 64 days for CT or MR438 and 67 days for RT alone). The effects of the combination of MR438 and RT on the NRP1 and CD15 expression were evaluated by immunohistochemistry analysis. Contrary to the heterotopic model, the number of NRP1 + cells did not change significantly after 1 week of treatment with MR438 or RT + MR438 (Fig. 5D). However, the number of CD15 + cells was significantly decreased in the MR438-treated tumors compared to the control tumors (Fig. 5E). To evaluate the eventual benefit of a longer period of treatment, experiments were performed with a 2 week treatment schedule (Fig. 6). This schedule did not appear to delay tumor growth (Fig. 6B) or to improve the median survival for the RT + MR438 group (Fig. 6C) compared to the RT group (74.5 days for RT + MR438, 79 days for RT alone 65 days for CTL, 59 days for MR438).

To observe the early effect of the 2 weeks of combined treatment, histological analysis was performed at D15 post-treatment (Fig. 6D and F). We observed a decrease of NRP1 + cells for the two groups treated with RT (Fig. 6D), which correlated with a significant decrease in CD15 + cells only in the RT + MR438 group (Fig. 6F). Nevertheless, this longer treatment time significantly increased the number of NRP1 + cells for RT group and RT + MR438 group at the end of the experiment (Fig. 6E). This increase also seems to be correlated with the increase in CD15 + cells (Fig. 6G). In addition, we studied the expression of Ki67 and type IV collagen (Col IV) and to investigate the effect of MR438 combined with radiotherapy on cell proliferation and vascularization on intracerebellum DAOY tumors respectively (Additional file 2: Figure S2 and Additional file 3: Figure S3). Concerning cell proliferation, treatment did not affect the percentage for Ki67 cells positive either after 1 or 2 weeks of treatment (Additional file 2: Figure S2). For tumor vascularization, after 1 week of treatment, no significance was showed for vessels number and area (Additional file 3: Figure S3B) but after 2 weeks of treatment the vessels area was significantly increase with RT + MR438 compared to CTL (Additional file 3: Figure S3C).

## Discussion

Radiation therapy (RT) is commonly accepted as one of the most essential treatments for medulloblastoma after total resection of the tumor. RT can improve survival and reduce recurrence but remains aggressive with severe cognitive and endocrinal long term side effects, especially for young patients [2]. Therefore, it is necessary to develop new therapeutic modalities, that are more adapted to the biological nature of this tumor to improve not only the outcomes but also the quality of life of young patients. Recent studies have concentrated on targeted therapies mainly related to signaling pathways of the main 4 molecular subgroups: WNT, SHH, Group 3 and Group 4 [8]. The WNT and SHH pathways are better understood [33], while subgroups 3 and 4 are considered non-WNT non-SHH subgroups. Patients in the 3 and 4 subgroups or SHH subgroup with TP53 mutation have a higher risk of recurrence and a lower median survival [34–36]. It is even more urgent to find new therapies for these subgroups. In this work, we envisaged targeting MB stem cells more specifically by using combined treatment with radiotherapy and a peptidomimetic inhibiting NRP1 (MR438).

We have previously shown that medulloblastoma stem-like cells could overexpressed NRP1 in vitro in relation to the expression of CSCs markers such as CD15 or CD133, and that inhibition of NRP1 induced a differentiated status of this specific tumoral population [29]. Moreover, many studies suggested that CSCs are involved in resistance to radiotherapy and recurrence [13, 37, 38] and it was shown that medulloblastoma stem-like cells were more radioresistant than differentiated medulloblastoma cells [17]. We observe that the compound MR438 can radiosensitize medulloblastoma stem cells in vitro. Nevertheless, medulloblastoma stem cells from DAOY cells which belong to SHH mutated TP53 subgroup, are more radio-sensitive than medulloblastoma stem cells from D283-Med or D341-Med cells considered from subgroups 3 and 4 which are wild-type for TP53. TP53 is one of the most mutated genes in cancer and known to have functional role in the modulation of DNA repair and cell death after radiotherapy. Usually, loss of p53 function is correlated with increased radiation resistance in medulloblastoma cell lines as well as in medulloblastoma patients [35]. Nevertheless, it has been recently shown that TP53 mutated DAOY cells are radio-sensitized by a arsenic trioxide while TP53 wild-type medulloblastoma cells stay non-sensitive to radiation [39]. In vitro radiosensitivity with MR438 could dependent not only on NRP1 expression but also to DNA repair mechanisms probably due to genetic alterations linked to medulloblastoma subgroups.

In vivo, MR438 was able to radiosensitize the DAOY tumors, which presented a high level of NRP1 positive cells as compared to other tumor models. It was previously shown by Nasarre et al. that an antagonist peptide of NRP1 can decrease tumor growth in a glioma model by directly targeting tumor cells or blood vessels cells [40]. But contrary to this work, we did not observe an anti-proliferative effect with the compound alone on differentiated tumor cells or a vascular effect through endothelial cells [31]. Similarly, in our work, no effect on proliferation and vascularization have been observed with the compound alone in vivo. MR438 downregulated the expression of NRP1 in vitro [29], probably through a process of internalization/degradation of the NRP1 receptor after binding. This NRP1 downregulation exists in heterotopic tumor but is less evident in intracerebral tumors probably to specific mechanisms of NRP1 regulation not yet elucidated. Moreover, tuftsin, which is a natural ligand of NRP1, did not obtain the same effect as MR438 due to rapid degradation of the peptide, contrary to the peptidomimetic MR438.

This role of NRP1 in the sensitivity of cancer stem-like cells has been recently shown in glioblastoma multiform treated with chemotherapy (temozolomide) by using knockdown models [41]. The use of MR438 also led to decrease the number of MB stem-like cells as well as the decrease of stem cell markers, which led to a delay of growth for DAOY tumor when is associated with RT, as well as an increase in mouse survival, and a slight increase in the D283-Med mouse survival with MR438 used alone. Similarly, knockdown of NRP1 showed an increase in the radiosensitivity of human non-small-cell lung cancer cells (NSCLC), not only in vitro, but also in vivo*,* perhaps via the VEGF-PI3K-NF-kappaβ pathway [42]. We have previously shown that MR438 bound specifically to NRP1 but not to VEGFR2 [31] and this binding led to a downregulation of PI3K/AKT et MAPK pathways [29].

The NRP1 inhibition efficiency associated with RT was evaluated in an orthotopic xenograft model to take into consideration the influence of the tumor microenvironment. The treatment combination showed a benefit in terms of tumor growth or median survival with a total dose of 10 Gy. Unfortunately, when the duration of treatment is increased (2 weeks), the therapeutic combination is less efficient than RT alone (total dose of 20 Gy) probably due to the aggressivity of radiotherapy. Therefore, our combined treatment must be optimized perhaps by a longer administration of MR438 before or after radiotherapy. Nevertheless, NRP1 expression is clearly modified by the association of MR438 and RT at a higher dose with an early decrease followed by a late increase in our SHH orthotopic model. NRP1 overexpression has been recently observed in survived subclones of cell line H1299 after a single high irradiation dose of 10 Gy by Tustsumi et al. [43], that could explain the increase of NRP1 positive cells through a clonal selection in tumors at the end of the experiment for the longer treatment. Interestingly, the expression of the CD15 stem cell marker decreased strongly after the administration of MR438 demonstrating that it can target specifically medulloblastoma stem cells in vivo. CD15 seems to be directly correlated with NRP1 expression as recently published for pediatric brain tumor patients by our group (For more detailed information on NRP1 and cancer stem cells in pediatric brain tumors see reference [44]). Although NRP1 appears to be connected to the expression of stem cell markers [29, 41], no clear mechanism of regulation of this pathway has been yet identified. Several response mechanisms can take place, and it will be interesting to explore the mechanism recently proposed that NRP1 inhibition appeared to regulate RAD51 expression through the VEGFR2-independent ABL-1 pathway, and then increased radiation sensitivity [45]. Several authors demonstrated that NRP1 depletion did not only affect cell proliferation but did affect other cell functions, such as cell differentiation [41, 46], which strengthens our therapeutic approach based on the targeting of medulloblastoma stem-like cells. To develop more effective therapeutics strategies in the treatment of medulloblastoma, further studies of the molecular mechanisms in NRP1 signaling in cancer stem cells is needed to elucidate the rebound of NRP1 positive cells observed with 2 weeks of treatment. We can speculate that the non-response with the 2 weeks treatment is possibly due to a selection of resistant tumor cells to NRP1 inhibition or to other mechanisms of escape involving vascularization which are well-known to targeted therapies promoting other signaling pathways.

## Conclusions

In conclusion, the inhibition of NRP1 via MR438 could increase the radiosensitivity of medulloblastoma stem-like cells, especially at low doses. Finally, this work highlights the interest in targeting NRP1 in association with low cumulative doses of radiotherapy to limit MB progression by targeting stem-like cell numbers. Further studies are needed to explore the mechanism of regulation of NRP1 in MB progression through stem-like cells.

## Supplementary Information

**Additional file 1**: ** Figure S1.** Effect of MR438 on Ki67 expression in heterotopic xenografts in nude mice, at the endpoint of the experiment.

**Additional file 2**: ** Figure S2. **Effect of MR438 on Ki67 expression on intracerebellum DAOY tumors at the endpoint of experiment, after one week and two weeks of treatment.

**Additional file 3**: ** Figure S3. **Effect of MR438 on the vascularization on intracerebellum DAOY tumors at the endpoint of the experiment, after one week and two weeks of treatment.

## Data Availability

All data generated or analyzed during this study are included in this published article [and its supplementary information files].

## Abbreviations

bFGF: Basic fibroblast growth factor
CSC: Cancer stem cells
CTL: Control
EGF: Epidermal growth factor
MB: Medulloblastoma
MS: Medullosphere
NRP1: Neuropilin-1
qRT-PCR: Quantitative reverse transcription PCR
RT: Radiotherapy
