# Supplementary figures and images for: NRP1 inhibition modulates radiosensitivity of medulloblastoma by targeting cancer stem cells
Source: Cancer Cell Int. 2022 Dec 1;22:377. doi: 10.1186/s12935-022-02796-4 (PMC9714111; doi:10.1186/s12935-022-02796-4)

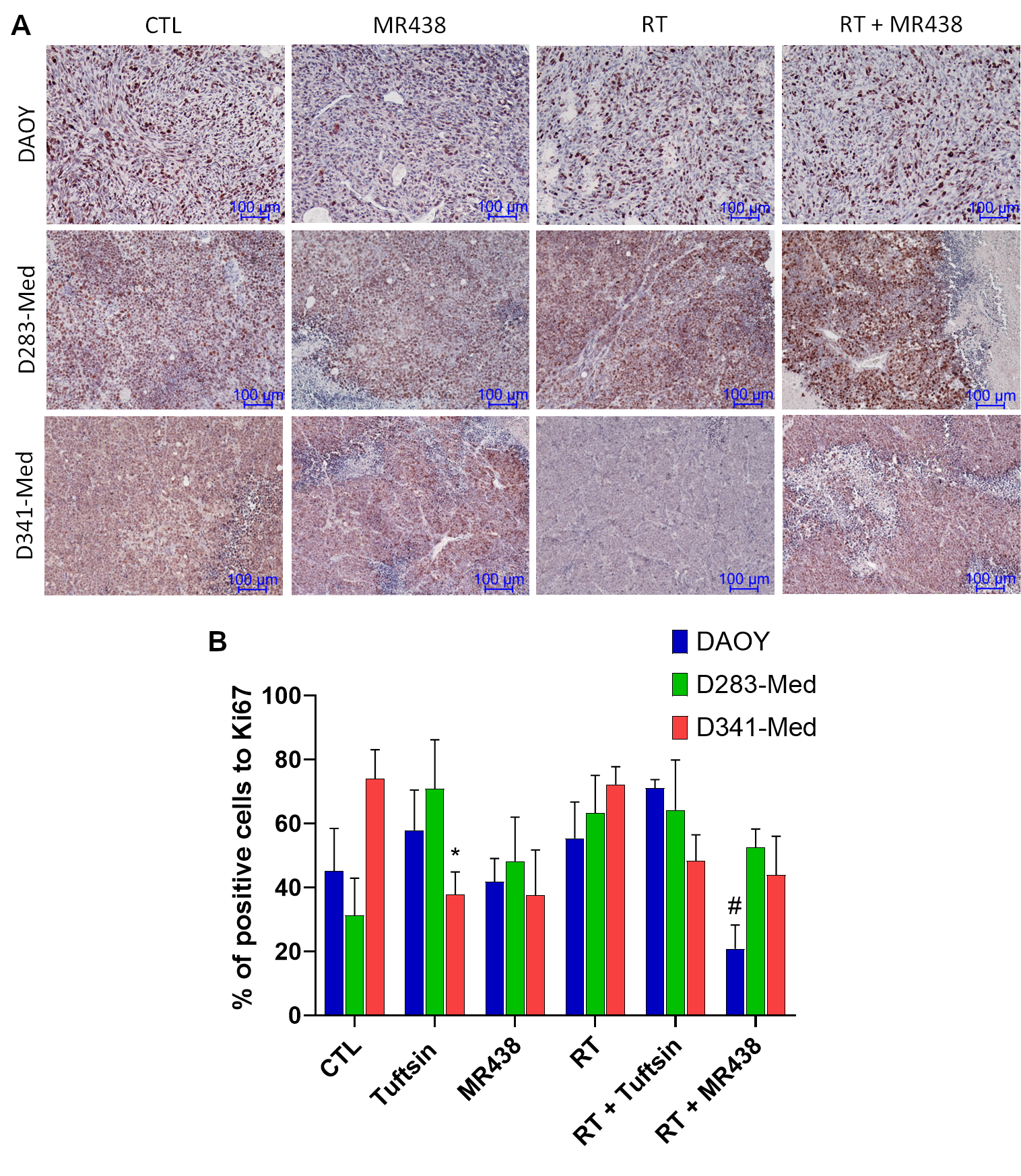

Supplement: Supplementary file 1 — Additional file 1: Figure S1. Effect of MR438 on Ki67 expression in heterotopic xenografts in nude mice, at the endpoint of the experiment. [file 12935_2022_2796_MOESM1_ESM.png]

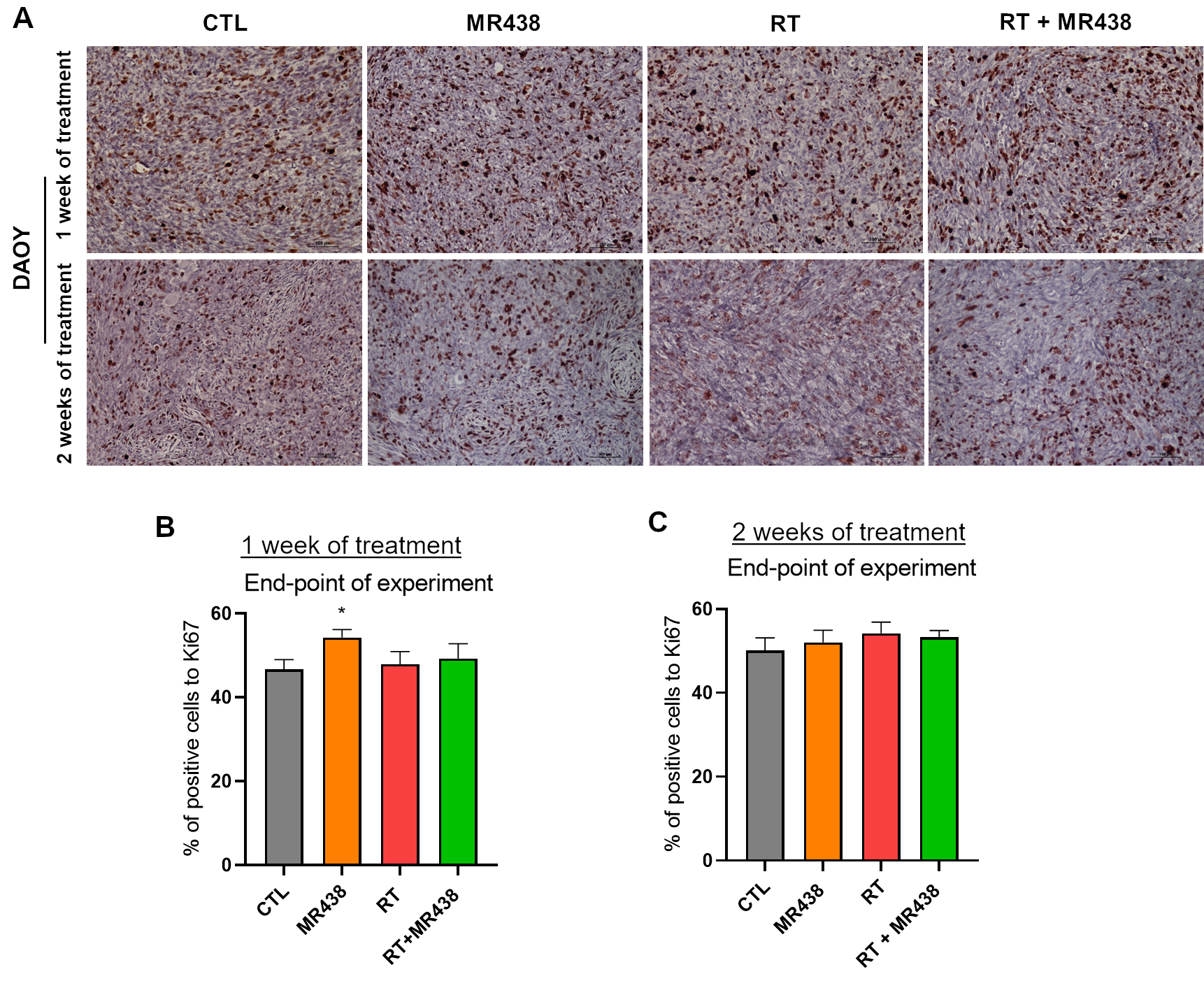

Supplement: Supplementary file 2 — Additional file 2: Figure S2. Effect of MR438 on Ki67 expression on intracerebellum DAOY tumors at the endpoint of experiment, after one week and two weeks of treatment. [file 12935_2022_2796_MOESM2_ESM.png]

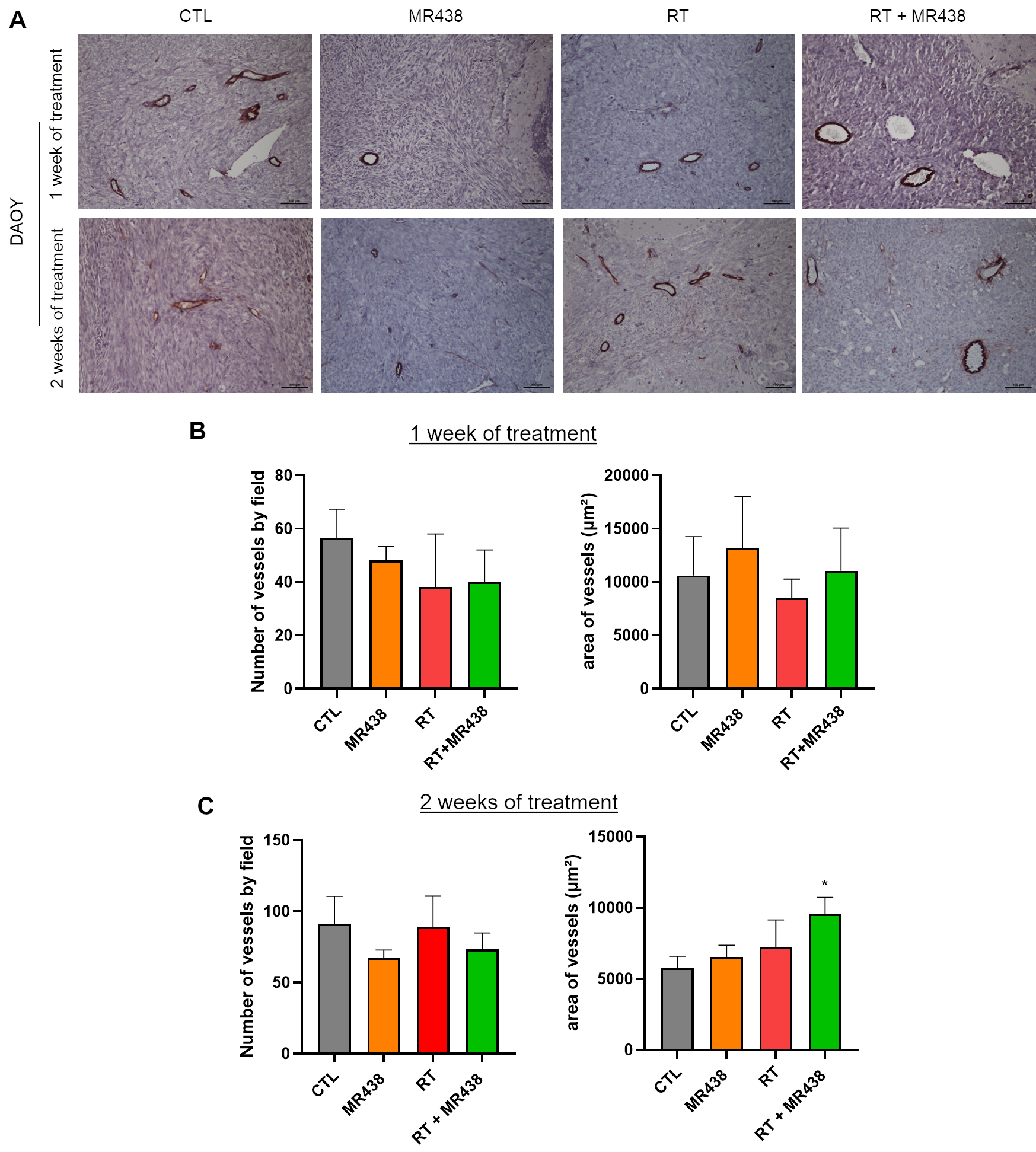

Supplement: Supplementary file 3 — Additional file 3: Figure S3. Effect of MR438 on the vascularization on intracerebellum DAOY tumors at the endpoint of the experiment, after one week and two weeks of treatment. [file 12935_2022_2796_MOESM3_ESM.png]
